# Next-Generation Antimalarial Drugs: Hybrid Molecules as a New Strategy in Drug Design

**DOI:** 10.1002/ddr.20345

**Published:** 2010-02

**Authors:** Francis W Muregi, Akira Ishih

**Affiliations:** 1 Department of Infectious Diseases, Hamamatsu University School of MedicineHamamatsu, Japan; 2 Centre for Biotechnology Research and Development, Kenya Medical Research Institute (KEMRI)Nairobi, Kenya

**Keywords:** malarial conjugates, trioxaquines and trioxolaquines, aminoquinolines, antimalarial resistance, malarial chemotherapy

## Abstract

Malaria is a disease that affects nearly 40% of the global population, and chemotherapy remains the mainstay of its control strategy. The global malaria situation is increasingly being exacerbated by the emergence of drug resistance to most of the available antimalarials, necessitating search for novel drugs. A recent rational approach of antimalarial drug design characterized as “covalent bitherapy” involves linking two molecules with individual intrinsic activity into a single agent, thus packaging dual-activity into a single hybrid molecule. Current research in this field seems to endorse hybrid molecules as the next-generation antimalarial drugs. If the selective toxicity of hybrid prodrugs can be demonstrated in vivo with good bioavailability at the target site in the parasite, it would offer various advantages including dosage compliance, minimized toxicity, ability to design better drug combinations, and cheaper preclinical evaluation while achieving the ultimate object of delaying or circumventing the development of resistance. This review is focused on several hybrid molecules that have been developed, with particular emphasis on those deemed to have high potential for development for clinical use. Drug Dev Res 71: 20–32, 2010. © 2009 Wiley-Liss, Inc.

**Table:** 

Strategy, Management and Health Policy				
Enabling Technology, Genomics, Proteomics	Preclinical Research	Preclinical Development Toxicology, Formulation Drug Delivery, Pharmacokinetics	Clinical Development Phases I-III Regulatory, Quality, Manufacturing	Postmarketing Phase IV

## INTRODUCTION

Chloroquine (CQ), a 4-aminoquinoline, has been the mainstay of malarial chemotherapy for much of the past five decades. The drug has several advantages including limited host toxicity ease of use, low cost, and effective synthesis. However, the use of CQ been eroded by development of resistance [Vangapandu et al., 2003; Kouznetsov and Gómez-Barrio, 2009]. Unfortunately, most AQ drugs are structurally related, and show cross-resistance [Foley and Tilley, 1998; Bloland, 2001]. Currently, artemisinin-based combination therapy (ACT) is the World Health Organization (WHO) golden standard against *Plasmodium falciparum* malaria, in which the regimen uses a double- or triple-combination therapy geared towards delay of resistance, or circumvents it altogether [Capela et al., 2009; Araújo et al., 2009; Maude et al., 2010]. Although no clinical resistance has been registered against artemisinins, recent reports from south-east Asia are increasingly pointing to tolerance, which may herald resistance against this class of drugs [Noedl et al., 2008]. Extensive spread of drug resistance involving classical antimalarials by *P. falciparum*, the most lethal strain of human malarial pathogen, necessitates a sustained search for promising compounds preferably with novel chemical structures and mechanism of action [Basco et al., 2001].

In the past two decades, only a few compounds belonging to a new class of antimalarial drugs, including aminoalcohols (mefloquine, halofantrine, lumefantrine), sesquiterpene trioxanes (artemisinin derivatives), and naphthoquinones (atovaquone) have been developed for clinical usage [Basco et al., 2001]. One of the challenges of future malarial chemotherapy is to develop compounds that are innovative with respect to the chemical scaffold and molecular target [Olliaro and Wells, 2009]. Many approaches to antimalarial drug discovery currently being deployed include optimization of therapy with available drugs including combination therapy, developing analogs of the existing drugs, evaluation of potent agents from natural products especially plants, use of compounds originally developed against other diseases, and evaluation of drug-resistance reversers (chemosensitizers) as well as new chemotherapeutic targets [Rosenthal, 2003; Kouznetsov and Gómez-Barrio, 2009]. Recently through rational drug design approach, single hybrid molecules with dual functionality and/or targets have been developed as novel antimalarial drugs. Some of these hybrid drugs have been demonstrated to be potent antimalarial agents, possessing no or minimum toxicity [Benoit-Vical et al., 2007; Coslédan et al., 2008]. However, so far none of these hybrid antimalarials have reached clinical application.

In malaria drug combination therapy, the current trend is to co-formulate two or more agents into a single tablet, termed as multicomponent drug (e.g., Coartem®, lumefantrine-artemether) as opposed to the traditional cocktail therapy, so as to improve patient compliance [Morphy and Rankovic, 2005]. However, based on the wide interest in the hybrid molecules as well as numerous encouraging efficacy and toxicity reports, the next generation of antimalarials may as well be hybrid drugs as opposed to multi-component ones. There are numerous advantages of employing hybrid molecules over multicomponet drugs in malaria therapy. Compared to the latter, hybrid drugs may be less expensive since, in principle, the risks and costs involved may not be different from any other single entity. Another advantage is that of the lower risk of drug–drug adverse interactions compared to multicomponent drugs. The downside, however, is that it is more difficult to adjust the ratio of activities at the different targets [Morphy and Rankovic, 2005].

Hybrid molecules can be classified as:

1. *Conjugates*, in which the molecular frameworks, that contain the pharmacophores for each target are separated by a distinct linker group that is not found in either of the individual drugs. Most conjugates contain a metabolically stable linker [Morphy and Rankovic, 2005].
2. *Cleavage conjugates* have a linker designed to be metabolized to release the two drugs that interact independently with each target.
3. *Fused hybrid* molecules have the size of the linker decreased such that the framework of the pharmacophores is essentially touching.
4. *Merged hybrids* have their frameworks merged by taking advantage of commonalities in the structures of the starting compounds, which give rise to smaller and simpler molecules [Morphy and Rankovic, 2005].

Quinine from Peruvian *Cinchona* trees provided the lead for the discovery and development of synthetic aminoquinolines, the most notable being CQ [Wang et al., 2007; Coslédan et al., 2008]. Likewise, the discovery of artemisinin from the Chinese herb *Artemisia annua* has served as a template for development of semi-synthetic artemisinins including artesu-nate and artemether, which are being used extensively in ACT against drug-resistant malaria [Maude et al., 2010]. The commercial availability of artemisinin (and hence its semi-synthetic derivatives) is limited by the fact that it is a natural product from *Artemisia annua*. Today, no fully synthetic peroxidic antimalarial drug has been made available for clinical application, which is unfortunate because of limitations associated with artemisinin semi-synthetics. The limitations include chemical (availability, purity, and cost), bio-pharmaceutical (poor bioavailability and limiting pharmacoki-netics), and treatment (non-compliance with repeated regimens and recrudescence) issues that limit their therapeutic potential [Vennerstrom et al., 2004; Perry et al., 2006]. As a result, extensive research into synthetic endoperoxide antimalarials drugs has been undertaken in the last 15 years to produce molecules that are structurally simpler and synthetically accessible with a projected low cost of goods [Sabbani et al., 2008]. Recently, fully synthetic peroxidic antimalarials are being developed, in which the pharmacophore of artemisinin is present within a 1,2,4-trioxalane, termed ozonide, rather than a 1,2,4-trioxane heterocycle of artemisinin [Uhlemann et al., 2007]. Synthetic 1,2,4-trioxolane derivatives being investigated as a new class of antimalarial peroxides offer the advantage of low cost of synthesis, improved biopharmaceutical properties, and excellent efficacy profiles compared with currently available artemisinin derivatives [Vennerstrom et al., 2004; Creek et al., 2008]. Other synthetic cyclic endoperoxides being explored include 1,2-dioxanes, 1,2,4-trioxanes, and 1,2,4,5-tetraoxanes, all of which retain the critical endoperoxide bond, which confers activity to artemisinins [Sabbani et al., 2008].

Recently, in a deliberate rational design of antimalarials acting specifically on multiple targets, several hybrid molecules have been developed in what has been termed “covalent bio therapy.” One instance is where a trioxane or trioxolane motif is covalently linked to a quinoline entity, to form new modular molecules referred to as *trioxaquines* or *trioxolaquines*, respectively [Araújo et al., 2009]. The quinoline nucleus has been a chemical reference of highly active antimalarial drugs for many decades and several effective drugs containing this entity including CQ, mefloquine, amodiaquine, and primaquine have been developed. The trioxanes and trioxolanes contain a peroxide bridge, which is essential for the high activity of artemisinin and its semi-synthetics including arte-mether, arteether, dihydroartemisinin, and artesunate. As the trioxane or trioxolane moiety is a potential alkylating agent after reductive activation by heme, and the 4-aminoquioline entity easily penetrates into infected erythrocytes and then interacts with heme, such modular molecules are expected to combine the dual activity of both fragments [Basco et al., 2001]. Trioxaquines are potent antimalarial drugs against both asexual and sexual malarial stages, and their potency is independent of CQ-sensitivity of the target parasite [Benoit-Vical et al., 2007].

## HYBRID MOLECULES WITH PROMISING CLINICAL PROSPECTS

### Fully Synthetic Peroxidic Molecules as Raw Materials for Hybrid Molecules

Endoperoxides currently in clinical use are semi-synthetics of the artemisinins, which include arteether, artemether, artelinic acid, and artesunate. Although these peroxides are highly potent with fast parasite clearance and broad parasite stage specificity, their major limitation is their short half-life, requiring regular dosing, which may lead to non-compliance and recrudescence [Gautam et al., 2009]. Therefore, these artemisinin semi-synthetic molecules are used in combination with long-acting antimalarial drugs in what is termed ACT [Maude et al., 2010]. Although the detailed mechanism of action of sesquiterpene lactone artemisinin, which contains a peroxide bond in the form of 1,2,4-trioxane heterocycle [Zhou et al., 2008], remains to be elucidated, it is widely accepted that its activity resides on the endoperoxide function. Basically, the cleavage of the endoperoxide-bridge by monomeric haem, which is released during parasites haemoglobin metabolism, forms carbon-centred free radicals, leading to alkylation of haem and other parasite biomolecules [Meshnick et al., 1993; Wei and Sadrzadeh, 1994; Robert and Meunier, 1998].

**Fig. 1:**
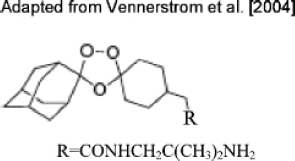
OZ277 (RBx-11160), a fully synthetic trioxolane and a potent peroxidic antimalarial.

Since the 1980s, there have been concerted efforts to shift from reliance on the first-generation artemisinin analogs, e.g., artemether and artesunate, and fully synthetic alternatives are being explored extensively. Although artemisinin semi-synthetics are highly potent and exhibit little or no cross-resistance with other antimalarials, they have poor bioavailability and pharmacokinetic properties, and are expensive since their availability depends on the availability of artemisinin from *Artemisia annua* [Ellis et al., 2008]. Concerted efforts geared towards development of fully synthetic alternatives, which retain the peroxide pharmacophore, have been applied for almost two decades although none of these fully synthetic molecules has reached clinical status. Efforts of the Vennerstrom group [Vennerstrom et al., 2004], among the pioneers in this venture led to the development of amine peroxides containing one peroxide bridge and later the 1,2,4,5-tetraoxanes and most recently the 1,2,4-trioxolanes (ozonides) and the clinical candidate OZ277 (RBx-11160) (Fig. 1). The latter is a fully synthetic trioxolane, a potent peroxidic antimalarial with a significantly different molecular structure from that of artemisinins [Vennerstrom et al., 2004; Creek et al., 2008]. To add to the superior antimalarial activity of the ozonide relative to conventional artemisinin semi-synthetics, oral dosing in rat models demonstrated a remarkable benign toxicological profile and lacked neurotoxicity due to a lack of accumulation in the brain [Vennerstrom et al., 2004]. Ellis et al. [2008] synthesized several 1,2,4,5-tetraoxanes that had antiplasmodial activity against *P. falciparum* in the range of 40–100 nM and were more stable than synthetic 1,2,4-trioxanes and -trioxolanes. Generally, 1,2,4-trioxanes, 1,2,4,5-tetraoxanes, and 1,2,4-trioxolanes were more active than the corresponding 1,2-dioxanes [Wang et al., 2007].

### Artemisinin-Based Hybrids Trioxaquines and Trioxolaquines

Trioxaquines are synthetic hybrid molecules containing two covalently linked pharmacophores (1,2,4-trioxane and an aminoquinoline), a concept referred to as “covalent biotherapy”, and thus possess a dual mode of action, namely heme alkylation with the trioxane entity, and heme stacking with the aminoquinoline moeity and inhibition of haemozoin formation [Loup et al., 2007; Coslédan et al., 2008]. Trioxolaquines are hybrid molecules similar to trioxaquines except that they contain a trioxolane motif, namely an ozonide, instead of a trioxane entity [Coslédan et al., 2008]. The first series of trioxaquines were potent against both CQ and pyrimethamine-resistant *P. falciparum* strains, and Benoit-Vical and co-workers developed the second series of trioxaquines, that were highly potent in vitro against both CQ-sensitive and -resistant *P. falciparum* isolates [Benoit-Vical et al., 2007]. The trioxaquines had more improved antimalarial activity than their individual fragments, indicating a potential additive/synergistic effect of the hybrids [Araújo et al., 2009]. Quinoline-endoperoxide hybrids have been developed with both semi-synthetic artemisinin derivatives as well as synthetic analogs, and possess remarkable in vitro antiplasmodial activity [Araújo et al., 2009]. Incorporation of 4-aminoquinoline or 9-aminoacridine (a component of mepacrine) into the final hybrid drug enhanced drug accumulation in the digestive vacuole of the parasite thus inducing a greater turnover of potentially toxic-free radicals by endoperoxide bioactivation.

**Fig. 2:**
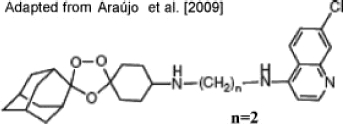
A 1,2,4-trioxolaquine, a potent hybrid antimalarial drug.

The potential advantage of both sets of compound lies in their capacity to target the parasite by two distinct mechanisms, thereby delaying or circumventing development of resistance [Araújo et al., 2009]. The synthetic peroxide hybrids (1,2,4-trioxalaquines) were generally more potent than their semi-synthetic (1,2,4-trioxaquines) counterparts. The most potent 1,2,4-trioxalaquine (Fig. 2) showed better *in vitro* antiplasmodial activity than either artemisinin or CQ against *P. falciparum* isolates [Araújo et al., 2009]. Although in some instances the hybrid molecule may lack a significant improvement of activity relative to the individual components of the hybrid, use of the hybrid drug would still be advantageous in several aspects. The derivatives may readily be converted into water-soluble salts making them suitable for oral or intravenous formulations. Also, when the peroxide component of the hybrid drug is “chemically consumed,” the residual aminoquinoline or aminoacridine constituent can still act as an efficient antimalarial, provided it is not covalently bound to the protein [Araújo et al., 2009].

**Fig. 3:**
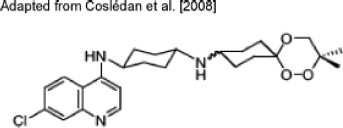
PA1103/SAR116242. The trioxaquine linker's cyclohexyl ring enhances its metabolic stability.

Coslédan et al. [2008] reported that from 2003-2006, 120 trioxaquines and trioxalaquines were developed and evaluated for antiplasmodial activity in vitro against both CQ-sensitive and -resistant *P. falciparum* isolates, giving IC_50_ values of 5–74 nM. Among the 120 compounds, 72 were evaluated for their ability to suppress the rodent parasite in mice, and 25 of the 72 molecules were considered for additional evaluation for in vitro absorption, distribution, metabolism, excretion, and toxicological evaluation. In the trioxaquine PA1103/SAR116242 (Fig. 3), the second cyclohexyl ring within the linker enhanced its metabolic stability relative to other trioxaquines with a linear aliphatic tether. The dual mode of action of PA1103/SAR116242 involved heme alkylation via the reductive activation of the trioxane entity, and heme stacking with the aminoquinoline moiety and inhibition of haemozoin formation [Coslédan et al., 2008]. The diastereoisomers of the trioxaquine were equipotent in their in vitro antiplasmodial activities against both CQ-sensitive and -resistant *P. falciparum* isolates (IC_50_ values = 7–24 nM), an additional gain to its potential as an antimalarial drug candidate. Both diastereoisomers had good oral bioavailability and were equipotent by p.o. in mice infected with *P. vinckei petteri*. The compound was also equipotent against CQ-sensitive and -resistant rodent parasites [Coslédan et al., 2008]. Humanized mice infected with human *P. falciparum* 3D7 (CQ-sensitive) and W2 (CQ-resistant) were either cured or had significant parasitaemia reduction when treated with the trioxaquine p.o. [Coslédan et al., 2008].

The observation that PA1103/SAR116242 was equally effective against both CQ-sensitive and -resistant *P. falciparum* isolates in humanized mice converges with the in vitro antiplasmodial outcome. In toxicological assays, the compound lacked mutagenic or clastogenic activity. The two separated diastereoisomers of the trioxaquine were equipotent in both in vitro and in vivo antiplasmodial assays, and displayed similar profiles in absorption, metabolism, and safety assays. The remarkable activity of PA1103/SAR116242 against both CQ-sensitive and -resistant *P. falciparum* strains, together with realization of dual mode of action (artemisinin-like and CQ-like), good bioavailability and low toxicity makes the molecule an attractive and promising candidate for a covalent bitherapy strategy [Coslédan et al., 2008].

Benoit-Vical et al. [2007] developed five second-generation trioxaquines (coded DU1301, DU1302, DU1313, DU1314, and DU2302), which they studied to reveal diverse biological activities and structural characteristics of this class of hybrid molecules. The in vitro antiplasmodial activities (IC_50_ values) of these compounds ranged from 4–32 nM and were independent of the CQ sensitivities of the *P. falciparum* isolates tested. These activities were similar to those of artesunate, the most potent drug of the artemisinin family available. To test the concept of dual activity of the molecules, the quinoline and trioxane precursors were tested individually and in combination, and their activities compared with that of the conjugates. Irrespective of the *P. falciparum* strain used, the trioxane entity alone had IC_50_ values ranging from 200–600 nM, while the IC_50_ values of the quinoline motif alone ranged from 120 nM to 2 μM. In combination in the same well, both entities had IC_50_ values ranging from 40–180 nM, whereas the trioxaquines' IC_50_ values ranged from 4–32 nM, implying that the link between both pharmacophores of the trioxaquines is essential for their activity. The diastereoisomers of these trioxaquines were equipotent against CQ-sensitive and -resistant strains of *P. falciparum*, implying that their target is achiral. In a 4-day suppressive test with mice infected with *P.v. petteri*, both DU 1301 and artesunate at doses of less than 30-mg/kg/p.o. qd threshold achieved a complete cure without any recrudescence. The trioxaquine, DU 1302, was an especially promising antimalarial agent since at a dose of 50mg/kg/day p.o., it was curative without any recrudescence in mice infected with the lethal strain *P. yoelii nigeriensis*, whereas 100-mg/kg/day p.o. artemisinin was required for a complete cure. DU 1302 also had gametocytocidal activity that was superior to that of artesunate, suggesting that the compound has potential to interrupt malaria transmission in a clinical situation. Furthermore, it lacked toxicity in an HCT cell line and in a mouse model. Synthesis of this trioxaquine prototype was also relatively simple, adding to its advantage as a future potent antimalarial drug [Benoit-Vical et al., 2007].

Basco et al. [2001] demonstrated that another trioxaquine derivative (DU-1102), was highly active against both CQ-sensitive and -resistant clinical *P. falciparum* isolates (mean IC_50_ = 43 nM). The responses to DU-1102 and chloroquine were, however, not correlated, suggesting an independent mode of action of the trioxaquine against the parasite.

### Artemisinin-Dipeptidyl Vinyl Sulfone Hybrids

**Fig. 4:**
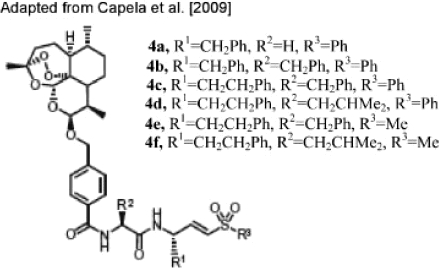
Artemisinin-dipeptidyl vinyl sulfone conjugates.

Cysteine proteases of the malarial parasite are of particular interest as therapeutic targets since they play major roles in parasite development. *P. falciparum* parasite expresses four cysteine proteases from the papain family known as falcipains, required for degradation of hemoglobin by erythrocytic malaria parasites, of which falcipain-2 and falcipain-3 are the most obvious as drug targets [Olson et al., 1999; Biot et al., 2007; Capela et al., 2009]. Several falcipain inhibitors including fluoromethyl ketones and vinyl sulfones inhibit parasite development in cultures by blocking the hydrolysis of host hemoglobin, and to cure mice infected with lethal *P. vinckei* infection [Olson et al., 1999]. Capela et al. [2009] have developed a series of endoperoxide-dipeptidyl vinyl sulfone hybrid molecules (Fig. 4) possessing dual activity of endoperoxide activation and falcipain inhibition. The vinyl sulfone moiety is covalently linked to the endoperoxide entity via the N-terminus, using a 4-hydroxymethyl-benzoic acid linker. The conjugate inhibited CQ-resistance *P. falciparum* isolate (W2) in the range 2-5 nM being more active than artemisinin and equipotent with artelinic acid. When screened against *P. falciparum* isolates with different phenotypes, FCR3 (atovaquone-resistant), 3D7 (CQ-sensitive), V1/S (CQ-and pyrimethamine-resistant), and D6 (CQ-sensitive, mefloquine-resistant), compounds **4a**, **4e**, and **4f** (Fig. 4) had superior activity when compared to CQ and artemisinin against all strains. Peptidyl vinyl sulfones are potent irreversible falcipain inhibitors, and hybrids that contained Leu-hPhe core inhibited falcipain-2 in the range 0.3–22 μM. The fact that the hybrid molecule falcipain inhibition was in the micromolar range implies that the endoperoxide pharmacophore contributed to the bulk of activity probably due to poor activity of the hybrids against falcipain-2 and/or their limited access to the food vacuole [Capela et al., 2009]. However, this served as a proof of concept, and future work in this regard is required to optimize the bi-functional molecule enzyme-binding capabilities.

Recently, Jones et al. [2009] reported artemisinin-acridine hybrids that had antitumour activity against various cell lines. Compared to dihydroartemisinin, these 1,2,4-trioxane-acridine hybrids had moderate antimalarial activity. While reporting on many artemisinin-based hybrids, the present discussion is in no way exhaustive, given the interest many researchers have shown in this field, not just for malaria chemotherapy but also for other diseases. There thus appear to be unlimited possibilities in which the artemisinin pharmacophore can be exploited in covalent bitherapy by linking it to other drug pharmacophores.

### Dual-Function Acridones

Recent developments in the design and synthesis of aminoquinoline ring-based hybrid molecules [Kouznetsov and Gómez-Barrio, 2009], especially bisquinolines [Foley and Tilley 1998], have been reported.

### Quinoline-Chemosensitizer Hybrid Molecules

Dual-function acridones refer to hybrid molecules based on the aminoquinoline ring, using similar previously described “one-drug, two-targets approach” as for artemisinin hybrid molecules. It is widely accepted that the parasite heme-detoxification process after hemoglobin metabolism is the primary target of quinoline drugs [Foley and Tilley, 1998]. The present evidence indicates that CQ resistance is directly associated with mutations in the gene encoding the digestive vacuole (DV) membrane protein of the *P. falciparum* CQ-resistance transporter (PfCRT) [Burgess et al., 2006]. The protein is predicted to function as an exporter of “metabolites” from the DV since it is a member of the drug/metabolite transporter superfamily [Bray et al., 2005]. Efflux of quinoline drugs results in reduced drug concentration at the target but does not alter the he me target itself [Kelly et al., 2009]. Thus, the target remains vulnerable, and the parasite is susceptible if the drug availability to the target can be restored. This is in contrast to drug resistance on the basis of protein target mutation, such as those that confer resistance against antifolates. Several compounds referred to as resistance reversers or chemosensitizers have been studied including verapamil and imipramine that reverse quinoline resistance [van Schalkwyk and Egan, 2006]. Unfortunately, the chemosensitizers have not been embraced for clinical use due to potency and safety concerns as well as their lack of intrinsic antimalarial efficacy. Thus, their combination with quinolines will represent monotherapy [Kelly et al., 2009].

**Fig. 5:**
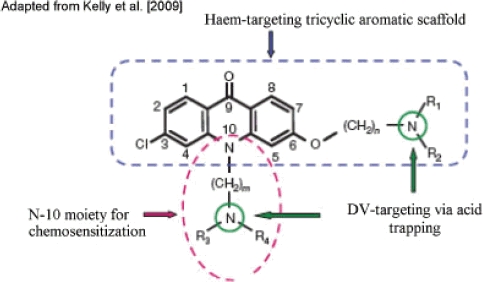
A generalized chemical structure of dual-function acridone derivatives. For compound T3.5: n,m = 2 and R_1_,R_2_,R_3_,R_4_ = CH_2_CH_3_.The rigid tricyclic acridone core promotes π-π stacking for haem binding. The side chain attachment at the central nitrogen atom provides a hydrogen bond acceptor needed for the chemosensitization function, and in conjunction with the side chain at position 6, facilitates accumulation in the digestive vacuole (DV) via acid trapping. [Color figures can be viewed in the online issue, which is available at http://www.interscience.wiley.com]

In an attempt to surmount these limitations, several attempts have led to the design of novel chimeric compounds modeled on the concept that mutations in the parasite DV membrane protein PfCRT lead to excessive efflux of CQ, and that protein activity can be inhibited by reversal agents [Burgess et al., 2006]. Kelly and co-workers [2009] incorporated the acridone phramacophore of the quinolines into a chemosensitization moiety to synthesize twelve hybrid molecules, with T3.5 [3-chloro-6-(2-diethylamino-ethoxy)-10-(2-diethylaminol-ethyl)-acridone] (Fig. 5) being the most promising antimalarial drug. The heme-targeting tricyclic group with an ionizable side chain promotes drug accumulation in the DV while a chemosensitization moiety at the N10-position is provided to counteract quinoline resistance. The side-chain attachment at the central nitrogen provides a hydrogen bond acceptor required for a chemosensitization function, a feature that is a well-established component of the pharmacophore for effective chemosensitizers [Kelly et al., 2007]. T3.5 had remarkable *in vitro* and *in vivo* (in mice) activity. T3.5 (100 mg/kg/p.o. qd) for 3 days diminished *P. berghei* parasitaemia by 95% with an initial highdose being curative (256 mg/kg/day, p.o.) with no overt toxicity in mice. The drug had significant synergistic interaction with several quinolines including CQ, amodiaquine, quinine, and piperaquine against multi-drug-resistant *P. falciparum* isolate, Dd2 [Kelly et al., 2009]. The synergy between T3.5 and quinine was also observed *in vivo* against patent infection with quinine-sensitive *P. yoelii*. Hybrid drug uptake and accumulation in the DV was successful, and that drug interacted with heme thus interfering with hemozoin formation [Kelly et al., 2009].

**Fig. 6:**
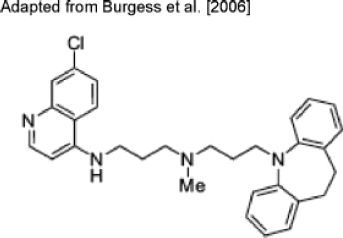
An aminoquinoline-imipramine hybrid molecule.

A similar multi-therapeutic approach was used by Burgess et al. [2006] to develop another hybrid molecule derived from the CQ skeleton and imipramine ring (Fig. 6). Imipramine is a well-known antidepres-sant, and is one of the better-studied PfCRT reversal agents known. The halogen core of CQ could effectively be linked to the imipramine moiety with no loss of heme-binding ability to yield a hybrid molecule that had remarkable antiplasmodial activity at low nanomolar levels against both CQ-sensitive (D6) and -resistant (Dd2) *P. falciparum* isolates. In mice, the molecule demonstrated high oral efficacy (99% suppression) against *P. chabaudi* without obvious toxicity [Burgess et al., 2006]. This approach is innovative in that if such molecules can clinically be demonstrated to enhance quinoline activity, then the traditional and unequalled advantages offered by 4-aminoquinolines (e.g., CQ) can be exploited in the fight against drug-resistant malaria. These advantages include low-cost and effective synthesis, limited host toxicity, short-course therapy, prophylactic and antipyretic effects. Furthermore, the strategy can be exploited to design new antimalarial chemotherapeutics to contol malaria. Thus, the design and synthesis of quinoline-chemosensitizers dual inhibitors or “double drugs” that would potentially inhibit hemozoin formation and another target within the malarial parasite should further be explored.

### Quinoline-Novel Target Based Hybrid Molecules

**Fig. 7:**
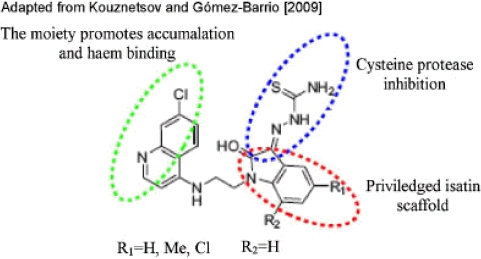
A 4-aminoquinoline-based isatin derivative. [Color figures can be viewed in the online issue, which is available at http://www.interscience.wiley.com]

**Fig. 8:**
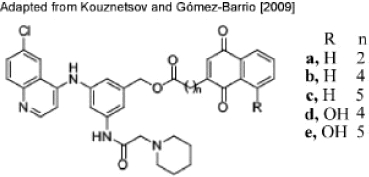
Aminoquinoline-glutathione reductase inhibitor conjugate molecules.

Cysteine proteases play critical roles in parasite that include but are not limited to general catabolic functions and protein processing [Sajid and McKerrow, 2002]. As stated earlier, the *P. falciparum* cysteine protease falcipains are essential for degradation of hemoglobin during erythrocytic parasite development. A new class of 4-aminoquinoline-based isatin derivatives (Fig. 7) was designed on the basis of a multi-therapeutic strategy. Isatin can easily be functionalized with the thiosemicarbazone moiety that could inhibit *P. falciparum-derived* cysteine proteases [Chiyanzu et al., 2005]. Thus, the quinoline entity could inhibit b-haematin formation whereas the isatin group inhibits *P. falciparum* cysteine proteases. Reports on the target compounds against both CQ-sensitive and -resistant *P. falciparum* strains, and against recombinant falcipain-2, demonstrated that the strategy is feasible. The hybrid molecules showed good in vitro antiplasmodial activity and inhibitory activity against falcipain-2, albeit modest. Thus, aminoquinoline-isatin hybrid molecules should be explored further as potential leads especially as regards the flexible ethylene linker and a thiosemicarbazone moiety. The ethylene linker increases the lipophilicity of the hybrid compound, which probably aids their passage through the parasite membranes to reach their presumed site of action, the acidic food vacuole [Chiyanzu et al., 2005; Kouznetsov and Gómez-Barrio, 2009]. Biot et al. [2007] synthesized chimeras of thiosemicarbazones and the new drug candidate, ferroquine, a new 4-aminoquinoline in which a ferrocenyl group is associated with CQ [Barends et al., 2007]. Hybrid molecules of this type had in vitro antiplasmodial activity against both CQ-sensitive and -resistant *P. falciparum* isolates, activity that was independent of parasite CQ-susceptibility [Biot et al., 2007]. Novel hybrids of phenolic Mannich bases linked to aminoquinoline fragment were synthesized by Chipeleme et al. [2007] and displayed significant antiplasmodial activity against *P. falciparum* isolate W2 (CQ-resistant) and inhibited cysteine protease falcipain-2. However, some hybrid analogs had activity against cultured parasites as well as falcipain-2, while some were either equipotent with CQ, or had improved activity (up to 3 times the activity of CQ). However, the ability of these compounds to inhibit falcipain-2 and their antiplasmodial activity against W2 were not correlated [Chipeleme et al., 2007]. Davioud-Charvet et al. [2001] developed 4-anilinoquinoline antimalarials based on the role reduced glutathione (GSH) plays in protecting *P. falciparum* from oxidative damage as well as in promoting heme detoxification [Atamna and Ginsburg, 1997; Becker et al., 2003; Wiwanitkit, 2006]. It was rationalized that increased GSH levels lead to increased CQ-resistance, and thus controlling levels of intracellular GSH by glutathione inhibitors could restore the efficacy of CQ and other 4-aminoquinoline analogues [Davioud-Charvet et al., 2001; Meierjohann et al., 2002]. Novel hybrid molecules based on aminoquinoline structures and 1,4-naphthoquinone ring were synthesized by esterification of naphthoquinolyl alkanoic acids with the alcohol amodiaquine derivative in the presence of dicyclohexylcarboiimide and a 4-(dimethylamino)pyridine [Davioud-Charvet et al., 2001]. It was anticipated that in the parasite, the molecule could be hydrolysed into two entities, with each product exerting its own action [Davioud-Charvet et al., 2001]. The compounds (Fig. 8) displayed good in vitro antiplasmodial activity against CQ-resistant *P. falciparum* isolate, FcB1R (ED_50_ = 23–56 nM) and remarkable low in vitro percent cytotoxicity against hMRC-5 cells (25–35 μM). The ether analog (Fig. 7c) affected the total glutathione content of the parasites [Davioud-Charvet et al., 2001]. These hybrid molecules of a quinoline ring–glutathione reductase inhibitor can serve as a model for development of novel hybrids and need to be further explored.

### Other Aminoquinoline-Based Hybrids

As it was earlier noted, the evidence from structure-activity relationship studies reveal that 4-aminoquinoline resistance does not involve any change to the target of this class of drugs but involves a compound efflux mechanism [Solomon et al., 2008]. Efforts are ongoing to restore 4 aminoquinoline sensitivity. These include synthesis of short chain analogs of 4-aminoquinolines, which are active against CQ-resistant strains of *P. falciparum*. However, the downside of the strategy is that after bioactivation the analogs are dealkylated, which lowers their lipid solubility. Recently, Solomon et al. [2008] have attempted to improve the lipophilicity of 4-aminoquinolines linking them with the cationic amino acids lysine and ornithine. The conjugates displayed activity against both CQ-sensitive and multidrug-resistant *P. falciparum* isolates in vitro and *P. yoelii in vivo*. Biot et al. [2004] developed several ferrocenic quinoline derivatives including a bis-quinoline and bisferrocene. One of the conjugates, the bis-quinoline 7-chloro-4-[4-(7-chloro-4-quinolyl)-7-ferrocenylmethyl-1,4,7-triazacy-clononan-1-yl]quinoline, showed potent antimalarial activity in vitro against the CQ-resistant *P. falciparum* isolate Dd2. This relatively new strategy involved incorportation of organometallocenic moiety of ferro-quine, which has cytotoxic properties into CQ pharma-cophore, which enables vectorizing the drug to the selected target [Biot et al., 2004]. Thus, the developed conjugate was expected to achieve vacuolar accumulation through pH trapping and haematin association conferred by 4-aminoquinoline, as well as lipophilic and redox properties conferred by ferrocenyl moiety [Biot et al., 2004]. This strategy underlines the potential of organometallic drug conjugates as novel antimalarials.

The 8-aminoquinoline, primaquine, is a tissue-schizontocidal drug that exerts its action against the primary and secondary tissue forms of the *Plasmodium* [Vangapandu et al., 2003]. Primaquine toxicity including inducing of hemolysis, limits its use in both prophylactic and therapeutic application. Also, its efficacy against asexual blood stages of the malarial parasite requires unacceptably high toxic doses, precluding its use in treatment of acute malaria as a blood schizontocide. Vangapandu et al. [2003] developed several “double prodrugs” or “pro prodrugs” of 8-quinolineamines so as to improve their bioefficacy. A pro prodrug is a derivative that undergoes two independent reactions to form the parent. The immediate prodrug must be a chemically reactive entity that undergoes a chemical conversion to release the bioactive parent drug under physiological conditions. However, this reactive prodrug is generated only after a biological step involving enzyme-catalyzed transformation of the chemically stable pro prodrug [Vangapandu et al., 2003]. The Vangapandu group hypothesized that 8-quinolinamine conjugates, where antimalarial analogs are attached to a carrier molecule (pro prodrug anchor), may carry the drug molecule to the site of action, where it may be released as the constituent prodrug by the action of esterases or reductases. The prodrug may subsequently be converted to the parent via a spontaneous independent chemical modification.

Many of the compounds from the pro prodrug series demonstrated promising in vivo antiplasmodial activity against drug-sensitive and -resistant rodent malarial parasites strains, confirming the hypothetical assumption that 8-aminoquinolines could be converted to biologically effective redox-sensitive and esterase-sensitive pro prodrug analogues. The most effective pro prodrug analog had promising activity against multi-drug-resistant *P.y. nigeriensis* in mice. The fact that “double prodrugs” displayed in vivo activity implies that endogenous enzymes present in the liver region achieved bioreactive and bioesterase activation to release the parent 8-quinolinamines [Vangapandu et al., 2003]. Rajić et al. [2009] developed primaquine conjugates in a further attempt to surmount its inherent toxicity and rapid biotransformation to carboxyprimaquine, which is devoid of antimalarial activity. Primaquine conjugates with glucosamine and two polymers of polyaspartamide type were synthesized. Some of the conjugates administered p.o. to mice infected with *P. berghei* parasite reduced parasitaemia significantly and prolonged mouse longevity, relative to the controls [Rajić et al., 2009].

## FUTURE PROSPECTS OF “COVALENT BITHERAPY” STRATEGY

The “Covalent bitherapy” strategy of drug design may find other future potential applications, which may include:

1. *Novel antimalarial drugs*: The principle of rational drug design of hybrid molecules has the potential to be extrapolated to other antimalarial drugs with different targets and/or mechanisms of action other than the ones discussed in this review. Trioxaquines offer better antimalarial activity than the individual components in double combination [Benoit-Vical et al., 2007]. This may imply that interaction of the pharmacophores of two drugs in the single-hybrid molecule is better than that of individual drugs in a combination. It is also possible that hybrid molecules will possess superior bioavailability and/or different mode of action from that of individual drugs in combination. This is especially useful in designing drugs such as the aminoquinolines (e.g., CQ), where resistance is not due to an altered target but failure to access the target. It will be interesting to evaluate whether the principle of “covalent bitherapy” can be exploited to develop modular hybrid molecules that restore activity of other dug class such as antifolates (e.g., sulfadoxine/pyrimethamine), which become ineffective due to resistance.
2. *Drug-delivery system*: As the search for novel drugs continues, more drugs with novel targets are being developed, but many of these do not reach clinical trials due to associated toxicity. Hybrid molecules are essentially prodrugs that aid in improving the efficacy and reducing the toxicity and other adverse effects of drugs by controlling their pharmacokinetic properties [Vangapandu et al., 2003]. Although some drugs may be toxic, their pharmacophores may not be as toxic, and it may be possible to develop safer drugs by covalently linking these pharmacophores with those of other drugs into synergistic conjugates. For instance, 5-fluoroorotate (FOA) is an active antifolate that targets thymidylate synthase (TS) of *P. falciparum* [Rathod et al., 1989, 1992; Rathod and Gómez, 1991; Gómez and Rathod, 1990; Muregi et al., 2009], but its clinical usage is curtailed by toxicity concerns. It is noteworthy that there is no antimalarial antifolate in clinical use that targets TS, and all antifolate antagonists in malarial chemotherapy only target either dihydrofolate reductase or dihydropteroate synthase of the parasite folate metabolic pathway [Nzila et al., 2005; Nzila, 2006]. Thus, clinical usefulness for FOA could be achieved by covalently linking it to another drug, ensuring safe delivery and selective toxicity to the malarial parasite cell, with no toxicity to the host cell. Primaquine, an 8-amino- quinoline, is a tissue schizontocide, which has serious side effects. Its toxicity limits its use in both prophylactic and therapeutic applications. It is known to induce hemolysis especially in glucose-6- phosphate dehydrogenase (G6PD)-deficient indivi duals [Vangapandu et al., 2003]. As described earlier, Vangapandu and co-workers [2003] demonstrated that 8-quinolineamines conjugates as well as their “double prodrugs” had promising in vivo activity in mice. If these compounds, in which basic pharmacophore is primaquine, are modified to improve their blood schizontocide activity, they have the potential to be used as broad-spectrum (tissue and blood schizontocides) antimalarial agents. Conjugation of drugs may, therefore, serve as a useful tool to improve drug solubility and stability, and prolong drug release, reduce doses, dosing intervals, and drug toxicity, as well as to achieve targetability [Rajić et al., 2009].
3. *Multiple(compound)-pharmacophoric hybrids*: Recently, a highly potent and promising ACT drug, a chlorproguanil hydrochloride-dapsone-artesunate (CDA, Dacart®) combination, was developed through collaborative work between WHO, MMV, and GSK. However, its Phase III trials and development were terminated prematurely because of toxicity concerns. Dapsone is known to induce hemolysis in G6PD-deficient individuals, who represent as much as 15% of the sub-Saharan Africa population, where over 90% of global malaria cases and deaths occur [Olliaro and Wells, 2009]. For the same reason, Lapdap® (chlorproguanil-dapsone) developed by WHO/GSK/University of Liverpool collaboration was withdrawn from the market. The potential of hybrid molecules linking more than two pharmacophores could be explored, since such compound hybrids may increase efficacy while abrogating the underlying toxicity. It would be interesting to see whether a hybrid molecule linking the three pharmacophores of CDA will circumvent toxicity.
4. *Tailor-made stage-specific hybrid molecules*: Covalently linking pharmacophores of antimalarial drugs that possess stage-specific action may have the potential of interrupting malaria transmission. For instance, drugs that target early asexual forms of the malarial parasite can be linked with the ones that target the late stages as well as the sexual forms (gametocytocides). Artemisinin semi-synthetics (e.g., artesunate), synthetic trioxaquines (e.g., DU1302), as well as primaquine have remarkable gametocytocidal activity [Pukrittayakamee et al., 2004; Benoit-Vical et al., 2007]. Artemisinins are also known to target early forms of the parasite, which is one of the main advantages of these drugs in ACT, reducing chances of development of resistance. In theory, therefore, it is possible to design hybrid molecules that integrate the various pharmacophores of such drugs, which will not only lower the parasite load effectively (thus reducing chances of resistance development), but also targeting sexual parasite forms can go a long way in interrupting transmission especially in malariaendemic areas.
5. *Elucidation of “drug mechanism of action” as well as “drug-resistance mechanisms”*: The molecular basis of action of most antimalarial drugs in clinical use is poorly characterized, much less the mechanisms involved in their resistance. While CQ action is thought to mainly involve interference with hemoglobin digestion in the blood stages of the malaria parasite life cycle [Foley and Tilley, 1998], the mechanism of action of artemisinins is a little controversial with various targets being reported although it is widely accepted that its activity is mediated via the endoperoxide function. It has been suggested that iron generates free radicals from artemisinins, which may lead to lipid peroxidation, protein oxidation, alkylation, and subsequent parasite death [Meshnick et al., 1993; Robert and Meunier, 1998]. Pandey et al. [1999] also reported that artemisinins exert their action in a similar manner to CQ, by interfering with the hemoglobin catabolic pathway and inhibition of heme polymerization. Eckstein-Ludwig et al. [2003] provided compelling evidence that after artemisinin activation by iron, it can inhibit *P. falciparum* sarco/endoplamic reticulum Ca^2+^-ATPase (SERCA) ortholog (PfATP6) outside the food vacuole. PfATPase is the only SERCA-type Ca^2+^ ATPase sequence in the *P. falciparum* genome. CQ-resistance has been implicated with elevated levels of drug efflux mediated by PfCRT, a member of the drug/metabolite transporter superfamily located in the intraerythrocytic parasite DV [Bray et al., 2005]. Although controversial, overexpression of the ATP-dependent drug transporter, P-glycoprotein, has also been implicated with CQ-resistance, and the *pfmdr1* gene product, PGh-1 (a typical member of P-glycoprotein family), is localized to the membrane of the parasite food vacuole [Foley and Tilley, 1998]. It may be postulated that hybrid molecules may aid in clarifying both the mechanism of action of drugs as well as in elucidating the mechanisms of resistance.

## CONCLUDING REMARKS

Hybrid molecules with dual functionality development and/or multitherapeutic strategies, which utilize new chemical entities with two (or more) different heterocyclic skeletons (pharmacophores), represent a valid and rational approach in design and development of novel antimalarials. These drugs have the potential to surmount the rapid development of resistance, enhance patient compliance, and reduce both the cost and the risk of drug–drug interactions. The strategy has the potential to restore efficacy of traditional drugs such as CQ, which have been rendered ineffective due to resistance, although the drugs had the advantage of the following benefits: affordable, effective synthesis, limited host toxicity, and short-course therapy. The current impetus in the search for fully synthetic peroxidic antimalarials as well as hybrid molecules at the laboratory level is yielding good results, and some of the drugs are now reaching the clinical trials stage. Fully synthetic peroxides may surmount the limitations of semi-synthetic artemisinins as they are superior in purity, cost, and biopharmaceutical properties. A fully synthetic peroxidic antimalarial, OZ277 (RBx-1160), is currently in development. Its combination with piper-aquine has been promising and Phase III clinical trials are underway in India [Uhlemann et al., 2007; Olliaro and Wells, 2009]. CDRI97/78, a trioxolane, is in Phase I trials. OZ439 is a next-generation ozonide compound, and its Phase I trials started in April 2009. It is being developed by the Medicines for Malaria Venture (MMV) in collaboration with Monash University and the University of Nebraska. Since most of the ozonides are structurally distinct from semi-synthetic artemisinins, it is expected that the problem of cross-resistance may not arise. This will be a great advantage especially now when some reports of artemisinin clinical tolerance are emerging mainly from the southeast Asia region, e.g., along the Thai-Cambodia border, which may herald a widespread global resistance against this class of drugs as was experienced with CQ [Olliaro and Wells, 2009; Noedl et al., 2008]. The trioxaquine, PA1103/SAR116242, originally developed by Palumed has been selected for full preclinical development as a drug candidate via the oral route, and is being developed with Sanofi-Aventis [Coslédan et al., 2008; Olliaro and Wells, 2009]. Although malarial chemotherapy may not be the silver bullet among the tools available for malaria control strategy, it remains the main arsenal against malaria since a vaccine is yet to be found, although one is in advanced clinical trials [Bejon et al., 2008; Maher, 2008]. It is, therefore, promising that novel antimalarial drugs are being developed, with some reaching clinical trials and with a high potential of clinical application.
